# Comparative effectiveness of angiotensin-converting enzyme inhibitors and angiotensin II receptor blockers in chemoprevention of hepatocellular carcinoma: a nationwide high-risk cohort study

**DOI:** 10.1186/s12885-018-4292-y

**Published:** 2018-04-10

**Authors:** Cheng-Maw Ho, Chih-Hsin Lee, Ming-Chia Lee, Jun-Fu Zhang, Jann-Yuan Wang, Rey-Heng Hu, Po-Huang Lee

**Affiliations:** 1 Department of Surgery and Department of Internal Medicine, National Taiwan University Hospital, Taipei, Taiwan; 2 College of Medicine, National Taiwan University, Taipei, Taiwan; 3 Division of Pulmonary Medicine, Wanfang Hospital, Taipei Medical University, Taipei, Taiwan; 4 Department of Pharmacy, New Taipei City Hospital, New Taipei, Taiwan; 5 Department of Internal Medicine, National Taiwan University Hospital, #7 Chung-Shan South Road, Taipei, 10002 Taiwan

**Keywords:** Hepatocellular carcinoma, Chemoprevention, High-risk cohort, Comparative effectiveness, Angiotensin-converting enzyme inhibitors, Angiotensin II receptor blockers

## Abstract

**Background:**

Research has revealed that angiotensin-converting enzyme inhibitors (ACEIs) and angiotensin II receptor blockers (ARBs) may prevent cancers such as hepatocellular carcinoma (HCC). The comparative chemopreventive effects of ACEIs and ARBs in high-risk populations with hepatitis B virus (HBV) or hepatitis C virus (HCV) infection have yet to be investigated.

**Methods:**

From 2005 to 2014, high-risk HBV and HCV cohorts of hypertensive patients without HCC history were recruited from three linked national databases of Taiwan, and were classified into two groups based on the ACEI or ARB exposure within the initial six months after initiating antiviral agent. Intergroup differences in clinical characteristics and duration of drug exposure within study period were evaluated. HCC-free survival was compared using the log-rank test. Multivariate Cox regression including time-dependent variables for the use of ACEIs or ARBs and other medications was applied to adjust for confounders.

**Results:**

Among the 7724 patients with HBV and 7873 with HCV, 46.3% and 42.5%, respectively, had an initial exposure to ACEIs or ARBs. The median durations of exposure were 36.4 and 38.9 months for the HBV and HCV cohorts, respectively. The median durations of ACEI or ARB use during study period between initial exposure and nonexposure groups were 41.8 vs. 18.3 months and 46.4 vs. 22.7 months for the HBV and HCV cohorts, respectively. No significant difference was observed in HCC risk within 7 years between the initial exposure and non-exposure groups. After adjustment for comorbidities, namely liver cirrhosis, diabetes mellitus (DM), and hyperlipidemia, and medications, namely aspirin, metformin, and statins, the hazard ratios (HRs) for ACEI or ARB exposure for HCC risk were 0.97 (95% confidence interval [CI]: 0.81–1.16) and 0.96 (0.80–1.16) in the HBV and HCV cohorts, respectively. In the HCV cohort, the increased HCC risk was associated with ACEI or ARB use in patients without cirrhosis, DM, and hyperlipidemia (HR: 4.53, 95% CI: 1.46–14.1).

**Conclusion:**

Compared with other significant risk and protective factors for HCC, ACEI or ARB use in the HBV and HCV cohorts was not associated with adequate protective effectiveness under standard dosages and may not be completely safe.

**Electronic supplementary material:**

The online version of this article (10.1186/s12885-018-4292-y) contains supplementary material, which is available to authorized users.

## Background

Hepatocellular carcinoma (HCC) is the third leading cause of cancer death worldwide and remains a major public health concern [1]. The established risk factors for HCC include chronic hepatitis B virus (HBV) and hepatitis C virus (HCV) infection, exposure to dietary aflatoxin, non-alcoholic steatohepatitis (NASH), alcohol-induced cirrhosis, obesity, smoking, diabetes, and iron overload [2]. The primary prevention strategy by HBV immunization is a milestone in reducing the incidence of HCC in children [3]. Secondary prevention by screening or surveillance in patients at a high risk of HCC is the strategy for reducing the associated mortality by providing interventions in the early stage of HCC [4, 5].

Chemoprevention is another attractive strategy for reducing the cancer incidence by administering drugs, typically for other reasons. For example, since angiotensin II stimulates neovascularization and could act as a growth factor for cancer, angiotensin-converting enzyme inhibitors (ACEIs) and angiotensin II receptor blockers (ARBs) could conceivably reduce cancer risk [6, 7]. Results of the study done by Chiang et al. suggested ACEI/ARB use lowers cancer risk [8]. On the contrary, a meta-analysis of randomized controlled trials suggests ARBs are associated with a modestly increased risk of new cancer diagnosis [9].Whether ACEIs or ARBs reduce cancer risk remains an issue to debate [7].

Though the chemopreventive effects of ACEIs and ARBs against HCC development and recurrence have been demonstrated in animal studies [10–12] and small clinical studies [13–15], large-scale study remains lacking. Numerous other medications have been reported to be associated with HCC chemoprevention, including HBV medications [16], interferons [17], metformin [18–20], statins [21, 22], aspirin [18], and nonsteroidal anti-inflammatory drugs [23, 24]. Similar as ACEI and ARB, some of the results are inconsistent and remain controversial [25, 26]. Considering that multiple risk and protective factors may coexist in patients at a high risk of HCC in real-world settings, the true comparative and competitive effectiveness of these chemopreventive drugs has not been comprehensively investigated.

The precise biological mechanisms underlying the protective effects of ACEIs or ARBs against cancer development is that the angiotensin I–VII levels increase during the inhibition of the ACE–angiotensin II–angiotensin II type 1 receptor (AT1R) axis, resulting in the activation of the Mas receptor and subsequent inhibition of cell proliferation and angiogenesis [27, 28]. Moreover, ACEIs or ARBs have been reported to reduce liver fibrosis in human studies [29, 30]. Further studies are warranted to determine whether these positive scientific findings can be used to extrapolate the efficacy of ACEIs or ARBs into clinical practice and translate this rationale into effective health intervention for high-risk populations [31]. The present study analyzed the comparative effectiveness of ACEIs and ARBs in the chemoprevention of HCC in high-risk cohorts.

## Methods

This was a retrospective study using nationwide cohorts of HBV and HCV patients with hypertension identified from a pseudonymied database. The primary outcome was HCC occurrence and the main exposure was the use of ACEIs or ARBs. The Institutional Review Board of National Taiwan University Hospital, Taipei, Taiwan, approved this study (NTUH REC: 201601007 W) and the need for informed consent was waived.

### Data acquisition

All original data were retrieved from three linked national databases covering the entire population of Taiwan from 2005 to 2014: Taiwan’s National Health Insurance Research Database (NHIRD), the Registry for Catastrophic Illness Patient Database (RCIPD), and the Cause of Death Database. Histological confirmation or typical imaging presentation of HCC is required for patients to be registered in the RCIPD [13].

### Cohort selection

Patients who received antiviral agents that fulfilled the reimbursement criteria for HBV (HBV medications) or HCV (interferons) therapy between 2007 and 2011 were identified, with the index date defined as the date of the first prescription. The reimbursement program for HBV and HCV therapy is basically a cost-effective policy in Taiwan and is strictly audited by definite evidence of diagnosis and laboratory data [32, 33]. Specialized case managers in each hospital guaranteed data accuracy. The enrolled population had higher risks of complications, including HCC, because of viral infection [33].

Among the enrolled patients, those with hypertension were further selected for analyses. The diagnosis of hypertension was confirmed according to the International Classification of Diseases, Ninth Revision, Clinical Modification (ICD-9-CM) codes (ICD-9-CM 401–405) in at least two outpatient visits within 120 days or at least one hospitalization and prescription of antihypertensive drugs (Anatomical Therapeutic Chemical [ATC] codes: C02, C03, C07, C08, and C09). Patients aged at least 20 years and fulfilling the aforementioned criteria from 2 years before to 6 months after the index date were included (Additional file 1: Figure S1). We excluded patients 1) diagnosed as having HCC (ICD-9-CM 155.0) before or within 6 months after the index date (to guarantee an induction period of at least 6 months after exposure to antihypertensive drugs), 2) who died within 6 months after the index date, or 3) those who received HBV medications or interferons from 2 years before to the index date.

### Demographic parameters

Demographic information, namely sex, age, liver cirrhosis, and comorbidities (diabetes mellitus [DM], hyperlipidemia, malignancies other than HCC, chronic obstructive pulmonary disease [COPD], end-stage renal disease [ESRD], transplantation, and alcohol consumption), was recorded. The diagnostic criteria for liver cirrhosis and hyperlipidemia are detailed in the Additional file 2 and those for other comorbidities were described in a previous study [34].

### Chemoprevention medications

The dosage and duration of the following drugs were recorded: 1) ACEI (ATC code: C09AA), 2) ARBs (ATC codes: C09CA and C09D), 3) low-dose aspirin for antiplatelet therapy (ATC code: B01AC06), 4) metformin (ATC code: A10BBA02), 5) statins (ATC codes: C10AA, C10BA, and C10BX), 6) HBV medications, and 7) interferons (see Additional file 3: Table S1 for the comprehensive list of drugs).

### Outcome measurements

The event date was the incidence of HCC confirmed by admission diagnosis (ICD-9-CM 155.0) or in the RCIPD. The patients were followed until death; withdrawal from the health insurance programs; or December 31, 2014. The date of death was obtained from the Cause of Death Database.

### Statistical analyses

Demographic characteristic and exposure to medications are shown separately for the HBV and HCV cohorts. Patients were divided into two groups based on the use of ACEIs or ARBs within 6 months after the index date, to demonstrate intergroup differences (initial exposure vs. initial nonexposure). The cumulative defined daily doses (DDDs) of medications were calculated in two groups. Data are expressed as mean ± standard deviation, median (interquartile range), or number (percentage), as appropriate. The Student t test or chi-squared test was used for intergroup comparisons. The time-to-event curves of different etiological groups were plotted using the Kaplan–Meier method and compared using the log-rank test.

Cox nonproportional hazards regression models with time-dependent covariates were used to study the association of medication use with the incidence of HCC in the HBV and HCV cohorts separately. The models were adjusted for the following covariates at the baseline (fixed in time): sex, age, income, liver cirrhosis, DM, hyperlipidemia, malignancies other than HCC, COPD, ESRD, transplantation, and alcohol consumption. Moreover, the following medications were adjusted as time-varying covariates according to the actual date of treatment initiation during the follow-up: ACEIs or ARBs, aspirin, metformin, and statins. Thus, only one observation was recorded per patient, and the status values of each medication were re-evaluated at every event time for each patient whose first medication prescription date was not a missing value and was earlier than the event time.

All statistical tests were two-sided at a significance level of 0.05, and all analyses were performed using SAS Version 9.4 (SAS Institute Inc., Cary, NC, USA).

## Results

### Patient population and baseline demographics

**Fig. 1:**
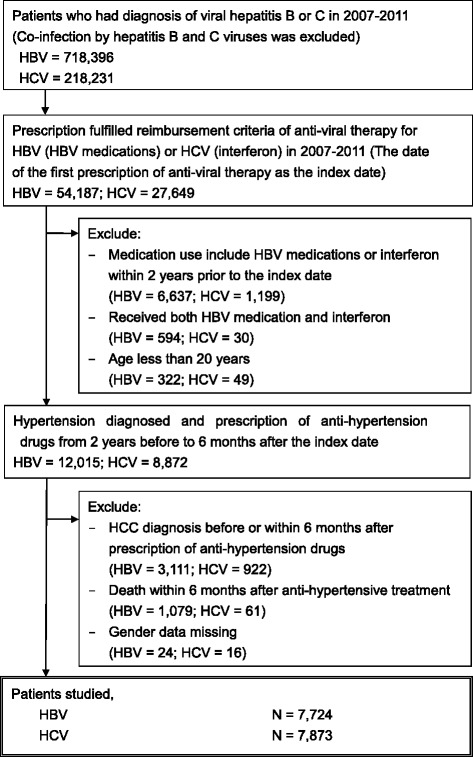
Flow diagram of patient selection

From 2007 to 2011, 54,187 and 27,649 patients with HBV and HCV, respectively, requiring antiviral therapy were identified. Antihypertensive drugs had been prescribed between 2 years before and within 6 months after the index date to 12,015 and 8872 adults with HBV and HCV, respectively. After the exclusion of patients with preexisting HCC, HCC diagnosis, or death within 6 months after the index date, 7724 patients with HBV and 7873 with HCV were included in the study cohort for analysis (Fig. 1).

Table 1 shows a comparison of the demographic characteristics based on whether patients were exposed to ACEIs or ARBs within 6 months (initial exposure vs. initial nonexposure) after the index date. In the HBV and HCV cohorts, 3575 (46.3%) and 3349 (42.5%) patients, respectively, were initially exposed to ACEIs or ARBs. The mean follow-up durations were 4.0 ± 1.7 and 4.6 ± 1.5 years for the HBV and HCV cohorts, respectively. HCCs were diagnosed in 7.1% (*n* = 552) of patients in the HBV cohort and in 6.4% (*n* = 503) of those in the HCV cohort. No patient was lost to follow-up. In both cohorts, the initial exposure group was significantly older, had more men, had diabetes and hyperlipidemia, and had less liver cirrhosis and alcohol consumption (Table 1). Additional file 4: Table S2 lists the use of other antihypertensive agents. Calcium channel blockers were the most common antihypertensive drugs in both HBV (61.4%) and HCV (64.1%) cohorts.

**Table 1:** Baseline characteristics of patients with HBV and HCV after exposure to ACEIs or ARBs within 6 months after the index date

	HBV patients				HCV patients		
Variables	All	Initial exposure	Initial non-exposure		All	Initial exposure	Initial non-exposure
n	7724	3575	4149		7873	3349	4524
Gender (male, %)	66.2	68.8^*^	63.9		49.3	51.8^*^	47.4
Mean age (years) (SD)	57.4 (11.3)	57.5 (11.2)	57.3 (11.5)		59.5 (9.1)	59.7 (9.3)	59.3 (9.0)
Liver cirrhosis (%)	78.9	77.8^*^	79.8		88.0	87.0^*^	88.7
Comorbidities							
DM (%)	36.6	43.8^*^	30.4		35.9	45.7^*^	28.6
Hyperlipidemia (%)	40.3	45.6^*^	35.7		31.3	36.4^*^	27.6
Other cancer (%)	24.8	21.1^*^	27.7		4.6	4.2	5.0
COPD (%)	11.4	11.3	11.5		12.0	12.5	11.7
End-stage renal disease (%)	1.7	1.7	1.6		1.2	1.3	1.2
Transplant (%)	2.9	3.1	2.7		0.6	0.9^*^	0.3
Alcohol use (%)	1.7	1.2^*^	2.2		0.7	0.4^*^	0.9
Mean follow up years (SD)	4.0 (1.7)	4.0 (1.6)	4.0 (1.7)		4.6 (1.5)	4.5 (1.4)	4.7 (1.5)
Last status							
HCC (n, %)	552 (7.1)	258 (7.2)	294 (7.1)		503 (6.4)	193 (5.8)	310 (6.9)
Death (n, %)	1282 (16.6)	508 (14.2)	774 (18.7)		261 (3.3)	115 (3.4)	146 (3.2)

*DM* diabetes mellitus, *COPD* chronic obstructive pulmonary disease, *HCC* hepatocellular carcinoma

^*^p < 0.05 between initial exposure and non-exposure groups

### Medications associated with HCC chemoprevention

The dosage and duration of antiviral medications for patients with HBV (HBV medications) and HCV (interferons) were used as recommended (Additional file 5: Table S3). Tables 2 and 3 presents the details of the medications potentially associated with HCC chemoprevention (ACEIs, ARBs, aspirin, metformin, and statins). In the HBV and HCV cohorts, the initial exposure groups had a significantly higher frequency than did the initial nonexposure groups in the prescription of the listed medications within 6 months after the index date (Table 2). The median cumulative DDDs are shown in Table 2. Table 3 shows the median DDDs and exposure duration of the medications of interests during the study period. Once administered, patients were provided with the standard dosage of these medications. The median durations of exposure to ACEIs or ARBs in the initial exposure and nonexposure groups were 41.8 versus 18.3 months in the HBV cohort and 46.4 versus 22.7 months in the HCV cohort, respectively (Table 3). In both cohorts, the initial exposure group had at least twice the length of time of exposure to ACEIs or ARBs compared with the initial nonexposure group.

**Table 2:** Medications for HBV and HCV patients in the initial 6 months after anti-hypertensive treatment (grouped by use of angiotensin converting enzyme inhibitors [ACEIs] or angiotensin receptor blockers [ARB] in the initial 6 months after index date)

		HBV patients			HCV patients	
Variables	All	Initial exposure	Initial non-exposure	All	Initial exposure	Initial non-exposure
n	7724	3575	4149	7873	3349	4524
ACEI (%)	13.4	28.9*	0	12.5	29.5*	0
Cumulative DDD^a^	168 (84–205)	168 (84–205)	0	168 (95–200)	168 (95–200)	0
ARB (%)	36.6	79.0*	0	32.5	76.5*	0
Cumulative DDD^a^	168 (87–198)	168 (87–198)	0	168 (98–196)	168 (98–196)	0
Aspirin (%)	18.8	23.2*	15.0	19.8	26.5*	14.9
Cumulative DDD^a^	308 (97–392)	309 (130–400)	284 (84–386)	330 (142–420)	336 (168–420)	309 (120–420)
Metformin (%)	19.0	24.4*	14.4	19.2	26.6*	13.7
Cumulative DDD^a^	84 (39–126)	88 (42–133)	75 (31–105)	89 (51–133)	90 (57–135)	84 (45–126)
Statin (%)	12.5	17.1*	8.6	5.9	8.4*	4.1
Cumulative DDD^a^	74 (37–112)	73 (37–112)	75 (30–112)	70 (37–112)	68 (37–112)	75 (35–112)

^a^Data are median (Q1-Q3). *p < 0.05 between initial exposure and non-exposure groups. *DDD* defined daily dose

**Table 3:** Medications for HBV and HCV patients in the study period (grouped by use of angiotensin converting enzyme inhibitors [ACEIs] or angiotensin receptor blockers [ARBs] used in the initial 6 months after index date)

		HBV patients			HCV patients	
Variables	All	Initial exposure	Initial non-exposure	All	Initial exposure	Initial non-exposure
n	7724	3575	4149	7873	3349	4524
ACEI/ARB						
DDD	1.0 (0.7–1.3)	1.0 (0.7–1.3)	0.9 (0.5–1.1)	1.0 (0.7–1.3)	1.0 (0.8–1.3)	0.9 (0.5–1.1)
Duration (months)	36.4 (12.5–50.0)	41.8 (22.2–53.6)	18.3 (3.0–35.9)	38.9 (18.4–53.1)	46.4 (36.1–57.9)	22.7 (6.8–38.6)
Aspirin						
DDD	1.6 (0.9–2.0)	1.7 (1.0–2.0)	1.5 (0.9–1.9)	1.6 (0.9–1.9)	1.7 (1.0–2.0)	1.5 (0.9–1.9)
Duration (months)	22.0 (3.3–44.5)	25.1 (4.9–45.5)	18.3 (1.5–42.4)	24.9 (4.9–46.9)	30.6 (7.1–48.6)	21.0 (3.1–45.3)
Metformin						
DDD	0.5 (0.3–0.7)	0.5 (0.3–0.7)	0.5 (0.3–0.6)	0.5 (0.3–0.6)	0.5 (0.3–0.7)	0.5 (0.3–0.6)
Duration (months)	26.7 (7.3–44.6)	29.5 (8.9–45.3)	23.6 (5.8–43.6)	35.5 (13.7–50.3)	37.3 (17.6–49.7)	31.4 (10.0–50.8)
Statin						
DDD	0.5 (0.3–0.8)	0.5 (0.3–0.8)	0.5 (0.3–0.8)	0.5 (0.3–0.7)	0.5 (0.3–0.7)	0.5 (0.3–0.7)
Duration (months)	23.3 (6.3–43.9)	25.7 (7.4–44.6)	20.7 (5.5–42.8)	20.0 (5.7–38.5)	22.5 (7.2–39.1)	17.4 (4.9–37.3)

Data are median (Q1-Q3). *DDD* defined daily dose

### Development of primary HCC

**Fig. 2:**
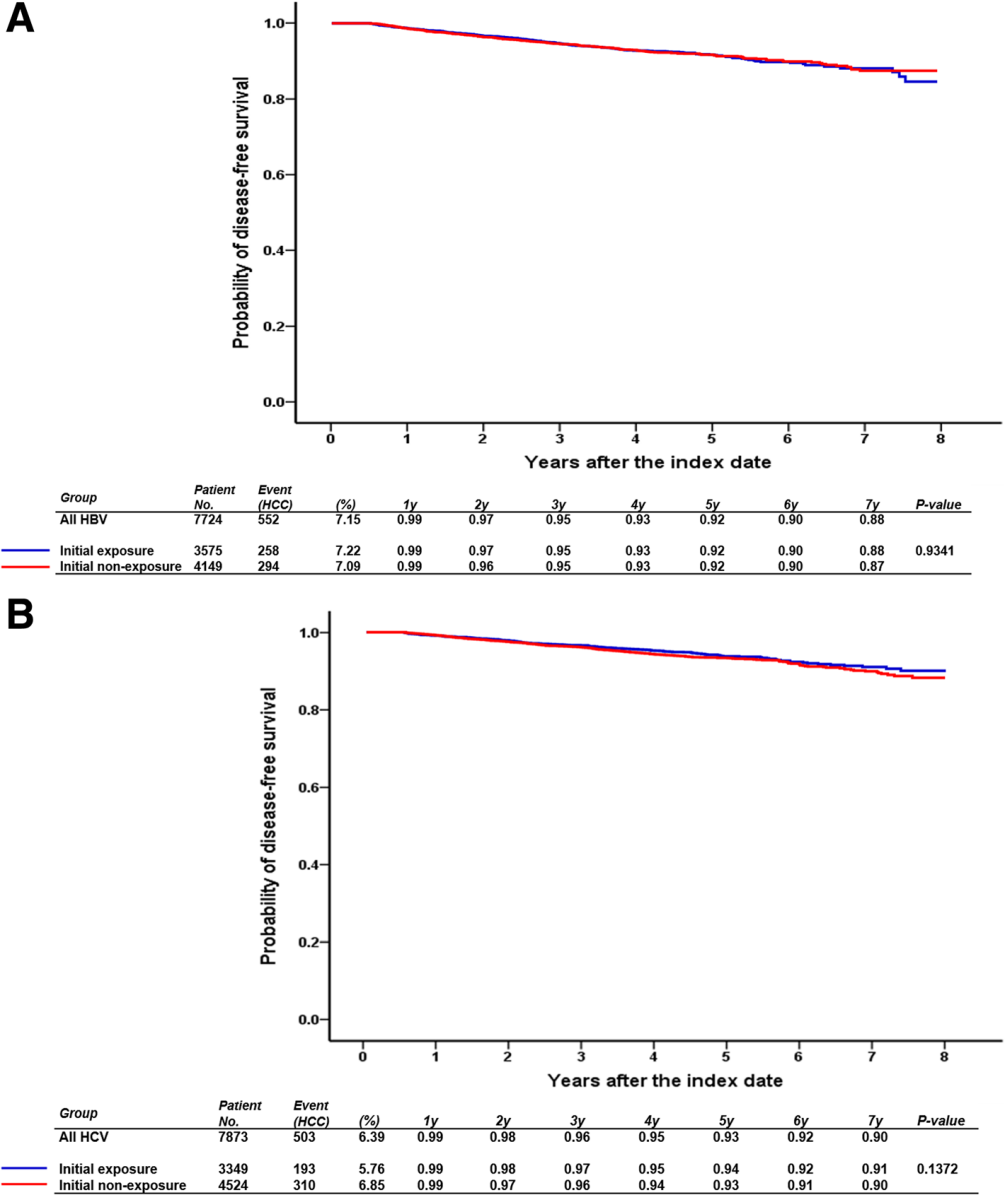
HCC-free survival for patients with HBV (**a**) and HCV (**b**) grouped according to the use of ACEIs and ARBs within 6 months after the index date

HCC-free survival curves are illustrated in Fig. 2. In the HBV cohort, the estimated 1-, 3-, 5- and 7-year HCC-free survival rates between the initial exposure and nonexposure groups were 99%, 95%, 92%, and 88% and 99%, 95%, 92%, and 87%, respectively, whereas in the HCV cohort, the corresponding rates were 99%, 97%, 94%, and 91% and 99%, 96%, 93%, and 90%. No significant difference was observed in HCC-free survival between the initial exposure and initial nonexposure groups in both HBV and HCV cohorts.

### Comorbidities as adjusted risk factors for primary HCC

Table 4 presents the effects of liver cirrhosis, DM, and hyperlipidemia on the risk of HCC. Notably, liver cirrhosis was a universal risk factor in both the HBV and HCV cohorts and in all subgroups. The adjusted hazard ratios (aHRs) were 2.40 (95% confidence interval [CI]: 1.74–3.32) and 1.76 (95% CI: 1.23–2.50) in the HBV and HCV cohorts, respectively. DM was a nearly universal risk factor in the HBV cohort, except in the subgroup of no liver cirrhosis, but not in the HCV cohort. Hyperlipidemia was a significant protective factor in the HBV cohort (aHR: 0.82, 94% CI: 0.67–1.00; Table 4a). The point estimate of the aHR for hyperlipidemia in the HCV cohort was similar (0.81), but with borderline significance (*P* = 0.057; Table 4b). In the HCV cohort, DM was a significant risk factor only in the hyperlipidemia subgroup; consistently, hyperlipidemia was a risk factor in the DM subgroup.

**Table 4:**
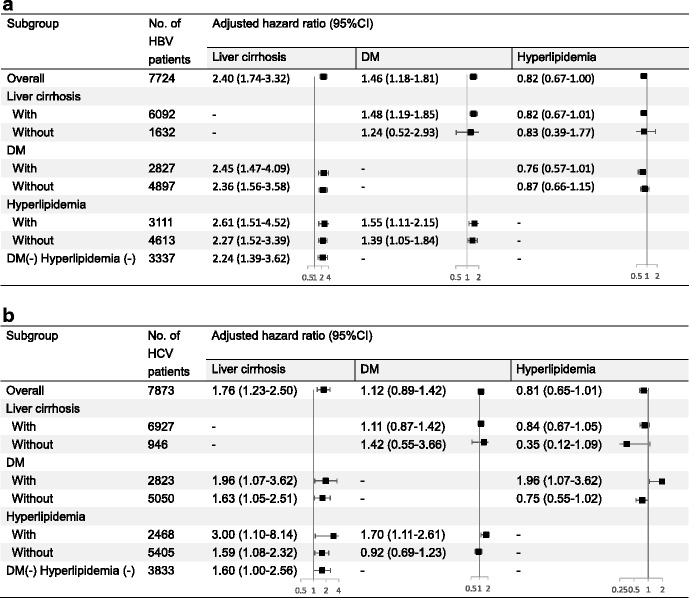
Effect of liver cirrhosis, DM, and hyperlipidemia on the risk of HCC in different subgroups of patients with HBV (a) and HCV (b)

*CI* confidence interval *DM* diabetes mellitus, *HBV* hepatitis B virus *HCV* hepatitis C virus

Model adjusted for age, sex, low economic income, other comorbidities (COPD, transplant, and other malignancy) and medications listed in Table 2

### Concomitant medications as adjusted risk factors for chemoprevention of primary HCC

Table 5 presents the effects of concomitant medications on the risk of HCC. ACEI or ARB use had a nearly neutral effect (aHR: 0.97, 95% CI: 0.81–1.16 in the HBV cohort; and aHR: 0.96, 95% CI: 0.80–1.16 in the HCV cohort). In the subgroup without any comorbidity (cirrhosis, DM, and hyperlipidemia), use of ACEIs or ARBs posed a significant risk in the HCV cohort (aHR: 4.53, 95% CI: 1.46–14.1, *P* = 0.009) and a potential risk in the HBV cohort (aHR: 1.65, 95% CI: 0.60–4.55, *P* = 0.330).

**Table 5:**
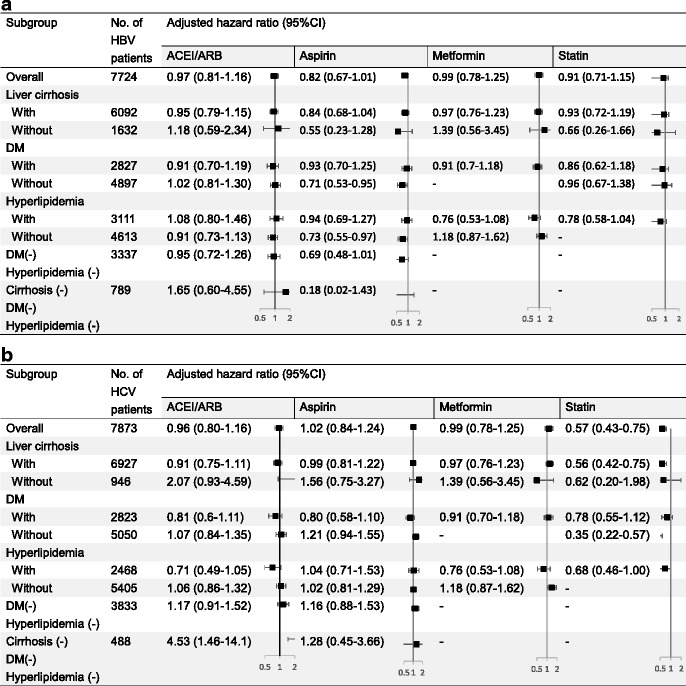
Effects of concomitant medications on the risk of HCC in different subgroups of patients with HBV (a) and HCV (b)

*CI* confidence interval *ACEI/ARB* angiotensin converting enzyme inhibitor or angiotensin receptor blocker, *DM* diabetes mellitus *HBV* hepatitis B virus, *HCV* hepatitis C virus

Model adjusted for age, sex, low economic income, other comorbidities (chronic obstructive pulmonary disease, transplant, and other malignancy), and medications listed in this table

Given that ACEI and ARB target at different nodes of renin-angiotensin system axis, their individual impact on the risk of HCC in different subgroups of patients with HBV and HCV were further analyzed separately (Additional file 6: Table S4). Because of the low number of HCC events (Additional file 6: Table S4), the adjusted effects of ACEI use were insignificant in all subgroups except two in the HCV cohort (no hyperlipidemia, aHR: 0.77, 95% CI: 0.59–1.00; and no cirrhosis, DM, and hyperlipidemia, aHR: 5.4, 95% CI: 1.91–15.2). Similarly, the adjusted effects of ARB use were insignificant in all subgroups in both HBV and HCV cohorts.

Aspirin had a significant protective effect in some subgroups (no DM or hyperlipidemia subgroups) of the HBV cohort (Table 5). Metformin was another neutral factor in the two study cohorts. Furthermore, statin was a significant protective factor in the HCV cohort (aHR: 0.57, 94% CI: 0.43–0.75) and most subgroups (cirrhosis, no DM, and hyperlipidemia), but without significance in the HBV cohort (*P* = 0.420).

## Discussion

The present nationwide cohort study of hypertensive patients with HBV and HCV infection who required antiviral therapy for alleviating the risk of primary HCC yielded four main findings. First, the estimated 7-year risks of primary HCC were 12% and 10% in the HBV and HCV cohorts, respectively. In addition, the risk did not significantly differ between patients exposed and not exposed to ACEIs or ARBs within 6 months after antiviral therapy. Second, after adjustment for comorbidities (including liver cirrhosis, DM, and hyperlipidemia) and time-dependent variables of medications (aspirin, metformin, and statins), ACEI and ARB use was not a significant predictor of primary HCC in the HBV cohort (aHR: 0.97, 95% CI: 0.81–1.16) and in the HCV cohort (aHR: 0.96, 95% CI: 0.80–1.16). Third, liver cirrhosis was a universal risk factor in both cohorts and in all subgroups. Finally, in the HCV cohort, ACEI or ARB use was associated with increased HCC risk in subgroups of patients without cirrhosis, DM, and hyperlipidemia.

The 5-year cumulative incidence of HCC in HBV patients with cirrhosis was 10%–17% [35]; the incidence was 11% in HCV patients with cirrhosis after a median follow-up of 6 years [36]. The present study yielded similar observations. Through regression modeling, competitive variables can be weight-based and compared. Among all comorbidities, liver cirrhosis was the strongest risk factor (aHR: 2.40, 95% CI: 1.74–3.32) in the HBV cohort, followed by DM (aHR: 1.46, 95% CI: 1.18–1.81); however, the effect of liver cirrhosis in the HCV cohort (aHR: 1.76, 95% CI: 1.23–2.50) was not this large. Because advanced cirrhosis is a relative contraindication for interferon therapy in patients with HCV [37], the use of interferon therapy as a patient selection criterion in this study may explain the significant but relatively low effect of liver cirrhosis on HCC development in the HCV cohort. Meanwhile, HBV patients with advanced liver cirrhosis or decompensation are candidates for HBV medication in the reimbursement program of Taiwan’s National Health Insurance. Therefore, these patients were recruited in this study. Nonetheless, the comparative effect of ACEIs and ARBs in the study cohorts was marginal and insignificant. These findings are important for developing policies on the selection of chemopreventive medications against HCC in high-risk groups.

Hepatocarcinogenesis is a complex multistep process in which many signaling cascades are altered, yielding a heterogeneous molecular profile [38]. Signaling pathways, such as the epithelial growth factor receptor (EGFR) and Ras, mammalian target of rapamycin, insulin-like growth factor receptor 1, hepatocyte growth factor and c-Met, Wingless, and angiogenesis, were, even if not entirely, involved in the complex and interactive system that formulates this hypervascular tumor entity [38]. The phenomenon of HCC heterogeneity not only exists in different tumors [39] but can also occur within a tumor [40]. The contemporary view of the involvement of the renin–angiotensin system in cancer, particularly HCC, might involve EGFR transactivation by AT1R and angiotensin II-independent, antiangiogenic effects of angiotensinogen [6, 41].The microenvironment in hepatocarcinogenesis becomes more complex in cases of viral infection and involves the transactivation of transcription factors and stimulation of inflammatory responses, thus resulting in oxidative damage, fibrosis, and genetic mutations [42]. Renin–angiotensin system signaling may have a potential role in hepatocarcinogenesis; however, the actual real-world comparative and competitive influences associated with other signaling pathways in HBV or HCV infection remain unknown. Limited human studies have reported the potential of ACEIs in reducing HCC recurrence after curative treatment [13–15]. Consistently, high gene expression of angiotensin-converting enzyme 2 in patients with HCC was associated with poor survival.

[43]. However, no human studies have investigated the primary prevention of HCC (targeting the initiation of hepatocarcinogenesis), probably because the recurrence rate of HCC is only adequately high in patients after curative resection [44]. Thus, the study endpoint could be reached in a manageable period.

Many possible reasons explain the gap between laboratory success and population insignificance. The dose–response relationship in vitro may not be applicable in standard dosage for hypertension. The doses recommended for cancer treatment are typically higher than the regular doses for treating non-cancer diseases. For example, the suggested dosing of everolimus, a mechanistic target of rapamycin inhibitor, in renal cell carcinoma is 10 mg per day, while it is 1.5 mg per day as an immunosuppressant in the setting of renal transplantation [45]. Therefore, there exists a translational inconsistency between laboratory and clinical practices. Our results can save the time and effort of researchers when conducting future human investigations.

Statistical results derived from a population-based database typically yield narrow point estimates and show statistical significance with ease, even when the absolute differences between groups are too small to have biological relevance (e.g., age difference of less than 1 year in adults). Therefore, the clinical significance may warrant further confirmation. The results may sometimes be misleading and induce population panic [46, 47]. We used multivariate Cox regression including time-dependent variables for medication use to carefully minimize the time-related bias associated with potential confounding medications in an observational study [25]. The neutral (or considered “negative”) effectiveness of ACEIs and ARBs in high-risk cohorts suggested that their association with the prevention of HCC, if it ever existed, was weak.

In human studies supporting the protective effects of ACEI or ARB [13–15], the outcome was HCC recurrence, which was different from the current study. Patients with history of HCC carry a much higher risk of HCC recurrence after treatment (and hence, a larger effect size) than those without history of HCC. Use of ACEI or ARB might, therefore, provide a “sufficient cause” for outcome prevention in the “super risky” population. Based on the results of this study, we estimated that a total of 34,058 and 19,274 event cases for HBV and HCV cohorts, respectively, are required to reach statistically significant. The effect sizes would be unrealistic since the current study already included the high-risk nationwide population at a largest and eligible scale.

In contrary, our study suggested that the use of ACEIs and ARBs was associated with an increased risk of HCC in HCV patients without cirrhosis, DM, and hyperlipidemia. ACEI exposure has been shown to be associated with breast cancer recurrence [48]. However, the mechanism underlying this observation and whether the impact is cancer-specific remain unknown [48]. As reported for ACEI, also ARB seem to increase new-cancer occurrence of lung [9], breast [49] and prostate [49], probably due to the unopposed effect on angiogenesis through angiotensin receptor 2 stimulation under angiotensin receptor 1 blockade [49]. Further study is warranted to confirm our finding and explore the pathophysiological mechanisms.

This study has some limitations. The NHIRD is a claims data source, which might lead to misleading findings if the study is solely based on it without validation. However, the selected cohorts (patients who received HBV and HCV therapy) in our study were strictly audited, and the outcome, HCC incidence, was retrieved from the RCIPD, a stringent certificate database. These confirmations minimized the bias of uncertainty in our study. We could not identify NASH patients accurately by ICD-9 classification, which is also a risk factor for HCC [50]. Future studies using the 10th version of diagnosis coding, in which there is a specific code for NASH, will help resolve this limitation. The other limitation of this study is that it did not consider some dietary [51, 52] and lifestyle-modifying factors, such as smoking [53], which might be involved in hepatic carcinogenesis and patient survival. Direct-acting antiviral therapy, which may reduce HCC incidence after HCV eradication [54], was not accessible in our cohorts. Furthermore, we did not investigate whether liver fibrosis can be resolved using ACEIs and ARBs.

## Conclusions

In conclusion, in the presence of other significant risk and protective factors for HCC, the use of ACEIs or ARBs in the HBV and HCV cohorts having a high risk of HCC was not associated with adequate protective effectiveness under standard dosages.

## Additional files

Additional file 1: **Figure S1.** Illustrative criteria of patient inclusion in the first step. (TIFF 127 kb)

Additional file 2: List of HBV and HCV medications, diagnostic criteria of liver cirrhosis, hyperlipidemia, and alcohol consumption. (DOCX 18 kb)

Additional file 3: **Table S1.** Anatomical Therapeutic Chemical codes and generic names of the chemoprevention drugs available in Taiwan during the study period. (DOCX 16 kb)

Additional file 4: **Table S2.** Antihypertensive medications (other than ACEIs and ARBs) for patients with HBV and HCV within 6 months after antihypertensive treatment, as grouped according to ACEI or ARB use within 6 months after the index date. (DOCX 20 kb)

Additional file 5: **Table S3.** Duration and amount of HBV medications and interferons in patients who received them in the study period, as grouped according to the use of ACEIs or ARBs within 6 months after the index date. (DOCX 17 kb)

Additional file 6: **Table S4.** Effects of ACEI and ARB on the risk of HCC in different subgroups of HBV and HCV cohorts. (DOCX 22 kb)

## Abbreviations

ACEI: Angiotensin-converting enzyme inhibitor
ARBs: Angiotensin II receptor blocker
AT1R: Angiotensin II type 1 receptor
ATC code: Anatomical Therapeutic Chemical code
CI: Confidence interval
COPD: Chronic obstructive pulmonary disease
DDD: Defined daily dose
DM: Diabetes mellitus
ESRD: End-stage renal disease
HBV: Hepatitis B virus
HCC: Hepatocellular carcinoma
HCV: Hepatitis C virus
HR: Hazard ratio
ICD-9-CM: International Classification of Diseases, Ninth Revision, Clinical Modification
NASH: Non-alcoholic steatohepatitis
NHIRD: National Health Insurance Research database
RCIPD: Registry for Catastrophic Illness Patient Database
