# Supplementary figures and images for: Comparative effectiveness of angiotensin-converting enzyme inhibitors and angiotensin II receptor blockers in chemoprevention of hepatocellular carcinoma: a nationwide high-risk cohort study
Source: BMC Cancer. 2018 Apr 10;18:401. doi: 10.1186/s12885-018-4292-y (PMC5891974; doi:10.1186/s12885-018-4292-y)

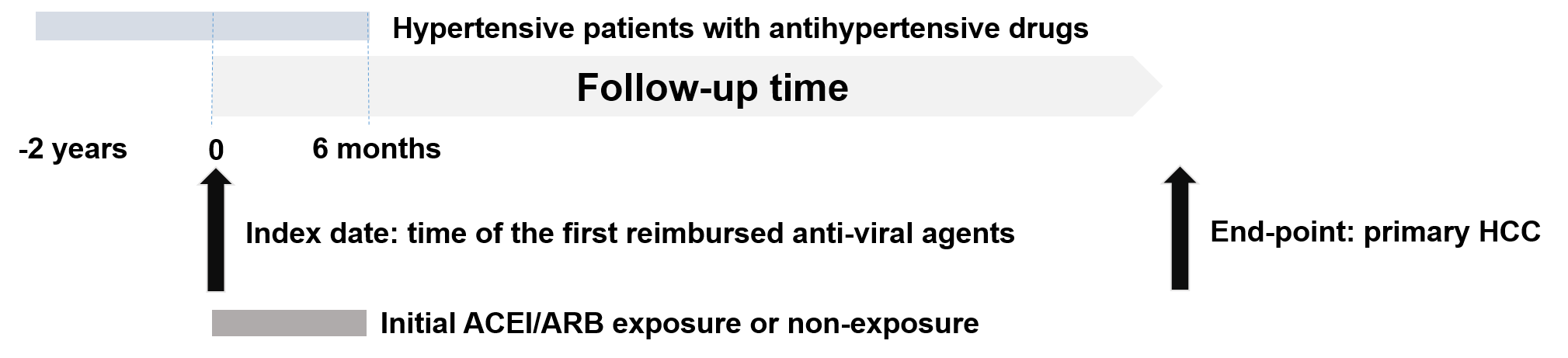

Supplement: Supplementary file 1 — Figure S1. Illustrative criteria of patient inclusion in the first step. (TIFF 127 kb) [file 12885_2018_4292_MOESM1_ESM.tif]
