# Supplementary material for: Comparative effectiveness of angiotensin-converting enzyme inhibitors and angiotensin II receptor blockers in chemoprevention of hepatocellular carcinoma: a nationwide high-risk cohort study
Source: BMC Cancer. 2018 Apr 10;18:401. doi: 10.1186/s12885-018-4292-y (PMC5891974; doi:10.1186/s12885-018-4292-y)
Supplement: Supplementary file 2 — List of HBV and HCV medications, diagnostic criteria of liver cirrhosis, hyperlipidemia, and alcohol consumption. (DOCX 18 kb) [file 12885_2018_4292_MOESM2_ESM.docx]

**Additional file 1**

HBV medications included lamivudine (J05AF05), adefovir (J05AF08), entecavir (J05AF10), telbivudine (J05AF11), and tenofovir (J05AF07). Interferon therapy included peginterferon alfa-2b (L03AB10, L03AB05, and L03AB60) and peginterferon alfa-2a (L03AB04, L03AB11, and L03AB61).

Liver cirrhosis was defined as (i) having an International Classification of Diseases, Ninth Revision, Clinical Modification (ICD-9-CM) diagnostic code 571.xx at admission or in the Registry for Catastrophic Illness patient database; (ii) ever receiving sclerosing therapy for varices (management codes: 47025A, 47025B, 47067B, 47078B, 71216B, 71217B, and 71218B); (iii) having an admission diagnostic code of spontaneous bacterial peritonitis (ICD-9-CM 567.23); or (iv) having complications of liver cirrhosis, such as hepatic coma (ICD-9-CM 572.2), portal hypertension (ICD-9-CM 572.3), hepatorenal syndrome (ICD-9-CM 572.4), or esophageal varices (ICD-9-CM 456.0, 456.1, and 456.2) within the 2 years before the index date or within 6 months after the index date.

Hyperlipidemia was defined as compliance with either of the conditions: (i) compatible diagnosis (ICD-9-CM 272.x and V77.91) and a prescription of lipid profile tests (management codes: 09095B, 260.61B, 09001C, 09043C, 09044C, 09092B, and 09004C) or (ii) a prescription of lipid-lowering agents (ATC codes: C10A and C10B).

Alcohol consumption was defined as having either of the conditions: compatible diagnosis (ICD-9-CM: 290.0–290.2, 291x, 303.x, 305, 305.0, 790.3, 980, E860.0, E860.1, E860.9, and V70.4) or prescription of laboratory tests (management code: 10807B).
