# Supplementary material for: Comparative effectiveness of angiotensin-converting enzyme inhibitors and angiotensin II receptor blockers in chemoprevention of hepatocellular carcinoma: a nationwide high-risk cohort study
Source: BMC Cancer. 2018 Apr 10;18:401. doi: 10.1186/s12885-018-4292-y (PMC5891974; doi:10.1186/s12885-018-4292-y)
Supplement: Supplementary file 3 — Table S1. Anatomical Therapeutic Chemical codes and generic names of the chemoprevention drugs available in Taiwan during the study period. (DOCX 16 kb) [file 12885_2018_4292_MOESM3_ESM.docx]

**Additional file 2: Table S1**. Anatomical Therapeutic Chemical codes and generic names of the chemoprevention drugs available in Taiwan during the study period

| **ATC** | **Generic name** |
| --- | --- |
| HBV medication (J05) | Adefovir, entecavir, lamivudine, telbivudine, tenofovir |
|  |  |
| Interferon (L03) | Interferon α-2A, 2B, Peg-interferon α-2A, 2B |
|  |  |
| Angiotensin converting enzyme inhibitor (C09A) | Captopril, enalapril, lisinopril, perindopril, ramipril, quinapril, benazepril, cilazapril, fosinopril, imidapril |
|  |  |
| Angiotensin II receptor blocker (C09C) | Losartan, eprosartan, valsartan, irbesartan, candesartan, telmisartan, olmesartan |
|  |  |
| Low-dose aspirin (B01A) | Aspirin |
|  |  |
| Metformin (A10BA02) | Metformin |
|  |  |
| Statin (C10) | Atorvastatin, ezetimibe, eluvastatin, lovastatin, pravastatin, rosuvastatin, simvastatin |
