# Supplementary material for: Comparative effectiveness of angiotensin-converting enzyme inhibitors and angiotensin II receptor blockers in chemoprevention of hepatocellular carcinoma: a nationwide high-risk cohort study
Source: BMC Cancer. 2018 Apr 10;18:401. doi: 10.1186/s12885-018-4292-y (PMC5891974; doi:10.1186/s12885-018-4292-y)
Supplement: Supplementary file 4 — Table S2. Antihypertensive medications (other than ACEIs and ARBs) for patients with HBV and HCV within 6 months after antihypertensive treatment, as grouped according to ACEI or ARB use within 6 months after the index date. (DOCX 20 kb) [file 12885_2018_4292_MOESM4_ESM.docx]

**Additional file 3: Table S2.** Antihypertensive medications (other than ACEIs and ARBs) for patients with HBV and HCV within 6 months after antihypertensive treatment, as grouped according to ACEI or ARB use within 6 months after the index date

|  | **HBV patients** | | |  | **HCV patients** | | |
| --- | --- | --- | --- | --- | --- | --- | --- |
| **Variables** | **All** | **Initial exposure** | **Initial non-exposure** |  | **All** | **Initial exposure** | **Initial non-exposure** |
| n | 7724 | 3575 | 4149 |  | 7873 | 3349 | 4524 |
| **ATC-C02** (anti-hypertensive) (%) | 10.1 | 11.8* | 8.6 |  | 8.4 | 9.8* | 7.4 |
| **ATC-C03** (diuretics) (%) | 37.8 | 38.0 | 37.7 |  | 21.5 | 25.8* | 18.3 |
| **ATC-C07** (β**-**blocker) (%) | 43.5 | 44.7 | 42.5 |  | 41.6 | 42.7 | 40.8 |
| **ATC-C08** (calcium channel blocker) (%) | 61.4 | 64.4* | 58.7 |  | 64.1 | 62.6* | 65.3 |

**p*<0.05 between initial exposure and non-exposure groups.
