# Supplementary material for: Comparative effectiveness of angiotensin-converting enzyme inhibitors and angiotensin II receptor blockers in chemoprevention of hepatocellular carcinoma: a nationwide high-risk cohort study
Source: BMC Cancer. 2018 Apr 10;18:401. doi: 10.1186/s12885-018-4292-y (PMC5891974; doi:10.1186/s12885-018-4292-y)
Supplement: Supplementary file 5 — Table S3. Duration and amount of HBV medications and interferons in patients who received them in the study period, as grouped according to the use of ACEIs or ARBs within 6 months after the index date. (DOCX 17 kb) [file 12885_2018_4292_MOESM5_ESM.docx]

**Table S3.** Duration and amount of HBV medications and interferons in patients who received them in the study period, as grouped according to the use of ACEIs or ARBs within 6 months after the index date

|  |  | **HBV patients** |  |  |  | **HCV patients** |  |
| --- | --- | --- | --- | --- | --- | --- | --- |
| **Variables** | **All** | **Initial exposure** | **Initial non-exposure** |  | **All** | **Initial exposure** | **Initial non-exposure** |
| n | 7724 | 3575 | 4149 |  | 7873 | 3349 | 4524 |
| **HBV medication** |  |  |  |  |  |  |  |
| DDD | 1.0 (0.6-1.0) | 1.0 (0.7-1.0) | 1.0 (0.5-1.0) |  | - | - | - |
| Duration (months) | 35.2 (17.2-46.3) | 35.3 (18.6-46.1) | 35.1 (16.4-46.4) |  | - | - | - |
|  |  |  |  |  |  |  |  |
| **Interferon** |  |  |  |  |  |  |  |
| DDD | - | - | - |  | 1.0 (0.9-1.6) | 1.0 (0.9-1.6) | 1.0 (0.9-1.6) |
| Duration (months) | - | - | - |  | 5.0 (4.6-7.2) | 5.0 (4.6-6.4) | 5.0 (4.6-8.4) |

Data are median (Q1-Q3)
