# Supplementary material for: Comparative effectiveness of angiotensin-converting enzyme inhibitors and angiotensin II receptor blockers in chemoprevention of hepatocellular carcinoma: a nationwide high-risk cohort study
Source: BMC Cancer. 2018 Apr 10;18:401. doi: 10.1186/s12885-018-4292-y (PMC5891974; doi:10.1186/s12885-018-4292-y)
Supplement: Supplementary file 6 — Table S4. Effects of ACEI and ARB on the risk of HCC in different subgroups of HBV and HCV cohorts. (DOCX 22 kb) [file 12885_2018_4292_MOESM6_ESM.docx]

**Table S4.** Effects of ACEI and ARB on the risk of HCC in different subgroups of HBV and HCV cohorts

| Cohorts and subgroups  (HCC event/No. of cases) | Ever using ACEI | | |  | Ever using ARB | | |  | Ever using either one | | |
| --- | --- | --- | --- | --- | --- | --- | --- | --- | --- | --- | --- |
|  | Event/No. | Adjusted HR  (95% CI) | P |  | Event/No. | Adjusted HR  (95% CI) | P |  | Event/No. | Adjusted HR  (95% CI) | P |
| **HBV (552/7724)** | 112/1791 | 1.01  (0.82-1.25) | 0.932 |  | 284/4443 | 1.00  (0.84-1.19) | 1.000 |  | 331/5056 | 0.97  (0.81-1.16) | 0.739 |
| Cirrhosis (511/6092) | 107/1399 | 1.06  (0.85-1.31) | 0.622 |  | 258/3518 | 0.97  (0.81-1.16) | 0.752 |  | 303/3988 | 0.95  (0.79-1.15) | 0.619 |
| No cirrhosis (41/1632) | 5/392 | 0.50  (0.19-1.29) | 0.150 |  | 26/925 | 1.41  (0.73-2.72) | 0.313 |  | 28/1068 | 1.18  (0.59-2.34) | 0.643 |
| DM (250/2827) | 54/768 | 0.90  (0.66-1.22) | 0.490 |  | 140/1801 | 0.96  (0.74-1.25) | 0.765 |  | 162/2044 | 0.91  (0.70-1.19) | 0.502 |
| No DM (302/4897) | 58/1023 | 1.13  (0.84-1.51) | 0.417 |  | 144/2642 | 1.03  (0.82-1.30) | 0.790 |  | 169/3012 | 1.02  (0.81-1.30) | 0.842 |
| Hyperlipidemia (203/3111) | 47/770 | 1.14  (0.82-1.60) | 0.436 |  | 118/1976 | 1.04  (0.78-1.39) | 0.788 |  | 137/2215 | 1.08  (0.80-1.46) | 0.626 |
| No hyperlipidemia (349/4613) | 65/1021 | 0.92  (0.70-1.22) | 0.570 |  | 166/2467 | 0.97  (0.78-1.21) | 0.814 |  | 194/2841 | 0.91  (0.73-1.13) | 0.392 |
| No DM/hyperlipidemia (215/3337) | 39/686 | 1.05  (0.73-1.49) | 0.806 |  | 94/1716 | 0.97  (0.74-1.28) | 0.848 |  | 112/1979 | 0.95  (0.72-1.26) | 0.733 |
| No cirrhosis/DM/ hyperlipidemia (19/789) | 0/170 | 0.00  (-) | 0.993 |  | 13/403 | 2.23  (0.81-6.10) | 0.120 |  | 13/473 | 1.65  (0.60-4.55) | 0.330 |
|  |  |  |  |  |  |  |  |  |  |  |  |
| **HCV (503/7873)** | 104/2222 | 0.83  (0.67-1.04) | 0.104 |  | 260/5054 | 1.02  (0.85-1.23) | 0.830 |  | 304/5699 | 0.96  (0.80-1.16) | 0.677 |
| Cirrhosis (470/6927) | 96/1993 | 0.80  (0.63-1.00) | 0.051 |  | 241/4449 | 1.00  (0.83-1.21) | 0.977 |  | 280/5018 | 0.91  (0.75-1.11) | 0.365 |
| No cirrhosis (33/946) | 8/229 | 1.75  (0.76-4.00) | 0.186 |  | 19/605 | 1.53  (0.74-3.16) | 0.251 |  | 24/681 | 2.07  (0.93-4.59) | 0.075 |
| DM (196/2823) | 47/947 | 0.79  (0.57-1.11) | 0.177 |  | 114/2050 | 0.96  (0.71-1.29) | 0.786 |  | 129/2286 | 0.81  (0.60-1.11) | 0.187 |
| No DM (307/5050) | 57/1275 | 0.86  (0.64-1.16) | 0.319 |  | 146/3004 | 1.07  (0.85-1.35) | 0.590 |  | 175/3413 | 1.07  (0.84-1.35) | 0.597 |
| Hyperlipidemia (123/2468) | 33/747 | 1.03  (0.68-1.56) | 0.898 |  | 63/1732 | 0.72  (0.50-1.05) | 0.087 |  | 75/1913 | 0.72  (0.49-1.05) | 0.086 |
| No hyperlipidemia (380/5405) | 71/1475 | 0.77  (0.59-1.00) | 0.048 |  | 197/3322 | 1.15  (0.93-1.42) | 0.191 |  | 229/3786 | 1.06  (0.86-1.32) | 0.578 |
| No DM/hyperlipidemia (258/3833) | 47/954 | 0.84  (0.61-1.16) | 0.293 |  | 124/2211 | 1.15  (0.89-1.48) | 0.296 |  | 150/2529 | 1.17  (0.91-1.52) | 0.225 |
| No cirrhosis/DM/ hyperlipidemia (19/488) | 7/103 | 5.40  (1.91-15.2) | 0.001 |  | 10/290 | 2.22  (0.86-5.70) | 0.098 |  | 15/324 | 4.53  (1.46-14.1) | 0.009 |

Model adjusted for age, sex, low economic income, other comorbidities (chronic obstructive pulmonary disease, transplant, and other malignancy), and medications (aspirin, metformin, and statin).

ACEI, angiotensin converting enzyme inhibitor; ARB, angiotensin receptor blocker; CI, confidence interval; DM, diabetes mellitus; HBV, hepatitis B virus; HCC, hepatocellular carcinoma; HCV, hepatitis C virus.
